# 

**Published:** 1897-11

**Authors:** 


### CONTENTS.

ORIGINAL ARTICLES: PAGE

Poisoning of Cattle by Andropogon Sorghum. By VET. CAP. H. T. PEASE, F.Z.S., F.R.M.S. . . . 679
Uniformity of State Regulations as to State Examining Boards. By W. HORACE HOSKINS, D.V.S. . . . 684
Address of Welcome of Major John J. McCann to the Members and Delegates at Nashville on Behalf of the State and the Tennessee Centennial. . . . 689
The Actual Cautery. By WILLIAM DOUGHERTY, D.V.S. . . . 692
Presentation of the Subject of State Control of Tuberculosis (Concluded). By JOHN M. PARKER, D.V.S. . . . 693

ABSTRACTS FROM FOREIGN JOURNALS:

Foot- and Mouth-disease . . . 702
The Central Association (in Austria) for the Erection of Public Slaughter-houses . 705
Treatment of Obstruction of the Œsophagus . . . 706
Iodide of Starch . . . 706
Tuberculosis in the Dog . . . 707
A Peculiarly Interesting Case of Indigestion of the Stomach . . . 707

SELECTIONS . . . 708

REPORTS OF CASES:

Record of a Few Cases of Abdominal Surgery. By E. MAYHEW MICHENER, V.M.D. 711
Four Interesting Cases at Cloverdale Stock Farm. By J. CURTIS MICHENER, V.S. 714
Ulceration of the Stomach of a Horse, and Chronic Glanders of a Mule. By W. E. A. WYMAN, V.S. . . . 716

EDITORIALS:

Be Up and Doing . . . 718
Always Give Credit Where Due . . . 718
The Same Thing from Different Points of View . . . 718
Necrology . . . 719

CONTROL WORK . . . 720

LEGISLATION . . . 723

AMONG THE COLLEGES . . . 724

SOCIETY PROCEEDINGS:

Massachusetts Veterinary Association—Tennessee Veterinary Medical Association—Keystone Veterinary Medical Association—New York State Veterinary Medical Society—Missouri Valley Veterinary Medical Association—New York County Veterinary Association—Veterinary Medical Society, University of Pennsylvania—Veterinary Medical Association of New Jersey—The Montreal Veterinary Medical Association—Ontario Veterinary Alumni of Massachusetts—Chicago Veterinary Society—California State Veterinary Medical Association—Pennsylvania State Board of Veterinary Medical Examiners . . . 725

PERSONALS . . . 737

THE OUTSIDE WORLD . . . 742

